# Knowledge, attitude, and beliefs toward group behavior therapy programs among male adults attending smoking cessation clinics, cross-sectional analysis

**DOI:** 10.1186/s12889-021-10924-4

**Published:** 2021-05-05

**Authors:** Shatha A. Alduraywish, Meaad F. Alnofaie, Balqes F. Alrajhi, Fatima A. Balsharaf, Sarah S. Alblaihed, Alaa A. Alsowigh, Wafa S. Alotaibi, Fahad M. Aldakheel

**Affiliations:** 1 Department of Family and Community Medicine, College of Medicine, King Saud University, Riyadh, Saudi Arabia; 2 Prince Sattam bin Abdulaziz Research Chair for Epidemiology and Public Health, College of Medicine, King Saud University, Riyadh, Saudi Arabia; 3 College of Medicine, King Saud University, Riyadh, Saudi Arabia; 4 Department of Clinical Laboratory Sciences, College of Applied Medical Sciences, King Saud University, Riyadh, Saudi Arabia

**Keywords:** Attitude, Behavioral therapy, Beliefs, Group therapy, Knowledge, Smoking cessation

## Abstract

**Background:**

Group therapy assists individuals in learning many behavioral techniques for smoking cessation and providing each other with mutual support. Group behavior therapy is not routinely provided as a modality of tobacco cessation assistance in tobacco cessation clinics in Saudi Arabia despite it is effectiveness. Therefore, this study aimed to assess the knowledge, attitude, and beliefs toward group behavior therapy programs among male adults who attend smoking cessation clinics and to identify the associated factors.

**Methods:**

A cross-sectional study was conducted with a targeted sample of 229 males aged 18 and above who were attending smoking cessation clinics. The participants were randomly selected. Data were collected using a paper-based questionnaire. One-way ANOVA and chi-square test were used for statistical analysis.

**Results:**

Results showed a high percentage of the study participants were in the age group of 21–40 years. Most of them were consuming 10–20 cigarettes per day. Around 79% of the participants had previous attempted to quit smoking. This study demonstrated a deficit in knowledge about group behavior therapy. The mean score for attitude and beliefs was 5.3 out of 11. Multiple factors influenced their attitudes and beliefs, such as previous attempts to quit smoking (*p*-value < 0.05) and the number of cigarettes used per day (*p*-value = 0.03). The knowledge was found to be affected by the level of education (*p*-value = 0.04).

**Conclusion:**

The study demonstrates a deficit in knowledge about group behavior therapy and it shows that the level of education was associated with the knowledge. Additionally, previous attempts to quit smoking and the number of cigarettes used per day, influenced the participants’ attitude and beliefs toward group behavioral therapy. Increase awareness about the role of group behavior therapy in smoking cessation is required before this method is implemented in the routine practice.

**Supplementary Information:**

The online version contains supplementary material available at 10.1186/s12889-021-10924-4.

## Background

Smoking is one of the leading public health concerns [1]. Globally, almost 23% of adults smoke tobacco products; this includes more than 1 billion males and 250 million females [2]. In Saudi Arabia, a recent study that assessed the prevalence of smoking among adults showed that the prevalence of cigarette smoking was around 21.4% in 2018 [3]. It has been shown that smoking is considered a significant risk factor for developing several chronic diseases such as pulmonary disease, heart disease, and lung cancer [4]. The World Health Organization recognized tobacco as the second leading risk factor for death worldwide [5]. Therefore, implementing smoking cessation strategies would assist in reducing the burden of these diseases.

Many approaches have been utilized for smoking cessation. The biopsychosocial approach has been proven to increase the rate of smoking cessation. This approach includes pharmacological and non-pharmacological interventions. Pharmacological treatment includes nicotine replacement therapy (NRT), Bupropion, and Varenicline (Champix/Chantix). The non-pharmacological interventions include considering the positive and negative environmental factors that might play a role in the effectiveness of treatment [6]. Several studies have shown the significant impact of combining pharmacological approaches with supportive intervention in successful smoking cessation [7]. One of these studies showed an increase in chance from 70 to 100% to quit smoking [8].

Additionally, smoking has a behavioral component related to physical addiction to nicotine. Behavioral intervention improved long-term smoking cessation. There are a variety of behavioral therapy interventions, including individual behavioral counseling, brief advice/interventions, telephone counseling, open-group forms of behavioral therapy, and closed-group forms of behavioral therapy. However, open-group forms of behavioral therapy are better and more cost-effective compared to the other types [9–11].

Previous studies showed that group therapy assists individuals in learning many behavioral techniques for smoking cessation and providing each other with mutual support [12–14]. A recent Cochrane review that assessed the group behavior therapy programmes for smoking cessation concluded that group therapy is considered superior than self-help and other less intensive intervention in smoking cessation. However, the review demonstrated lack of evidence for assessing the effectiveness for group therapy compared to intensive individual counselling [12]. Another review that assessed the usefulness and advantages of group behavior therapy among smokers, conclude that group intervention should be incorporated the management plan for smoking cessation wherever possible, because it is generally more efficient that individual intervention [13].

A study from Canada in 2002 aimed to assess the efficacy of other partner support groups in smoking cessation; they found that people who received partner group support had a higher cessation rate [15]. Another randomized clinical trial conducted in China studied the effectiveness of group intervention in smoking cessation among smokers and found that group intervention was highly effective compared to the control group [16]. In addition, behavioral counseling was found to be highly effective therapy [12].

As part of Saudi Vision 2030, the government of Saudi Arabia is determined to enhance the quality of preventive and therapeutic healthcare services. The recent prevalence of tobacco uses and its consequences are among the major public health concerns in Saudi Arabia. Tobacco control is urgently needed in the country due to increasing in the number of smokers and its related deaths. It has been estimated that around 70,000 Saudis die from smoking-related diseases every year [17].

The Ministry of health in Saudi Arabia has established the Tobacco Control Program in 2002. This program offers several services related to all aspects of smoking awareness, its harms and methods to combat it. Additionally, it provides a series of smoking cessation clinics located in many cities around the country [18]. These clinics equipped with trained personnel, to offer free consultation as well as provision of free pharmacological treatment. Group behavior therapy is not provided as a modality of tobacco cessation assistance although it has been shown to be effective in previous studies. Understanding the knowledge, attitude and beliefs of people attending the smoking cessation clinics toward group behavior therapy would guide the decision maker to integrate this method in the smoking cessation services.

Since the application of group behavior therapy programs in Saudi Arabia is lacking, the aim of the current study was to understand the knowledge, attitude, and beliefs toward group behavior therapy programs and its associated factors.

## Methods

### Study design and study participants

A cross-sectional study was conducted in five governmental smoking cessation clinics from different areas in Riyadh, Saudi Arabia. These clinics are Aldiryah, Alezdehar, Alrowdah, Alaziziyah neighborhoods, and Imam Abdulrhman Alfaisal Hospital. This study was carried out from November 2019 to March 2020.

The inclusion criteria were as follows: adult males who attended smoking cessation clinics planning to quit smoking or follow-up regarding their smoking cessation treatment. The females were excluded from this study because the prevalence of Saudi female smokers was estimated to be around 1.5% according to a national health survey [19]. Additionally, women rarely attended smoking cessation clinics. The participants were selected randomly through a simple random sampling technique using a computer-generated patients’ list in the smoking cessation clinics to minimize bias.

### Sample size estimation

As there were no previous studies that assessed the knowledge, attitude, and beliefs toward group behavior therapy programs, a pilot study was conducted to estimate the sample size. Results from this pilot study showed that the level of knowledge was approximately 42.2%.

Using the single proportion formula ($n={Z}^2\alpha\ \frac{P\left(1-p\right)}{d^2}$), the estimated sample size for the study was 191 smokers, considering the 20% non-response rate out of a total of 229 smokers.

### Data collection

The data were collected using paper-based questionnaires distributed randomly to the people attending the smoking cessation clinics. The questionnaire was developed using previously published information [13, 20]. It was reviewed by two experts in the field, MA (Pulmonology Consultant) and MG (Family Physician), both are tobacco treatment specialists. The questionnaire included four sections: the first section contained socio-demographic information (age, economic status, and educational level). The second section focused on tobacco use behavior (previous attempts to quit, number of cigarettes per day, duration of tobacco use). The third section focused on knowledge about group behavior therapy programs. The fourth section was about attitudes and beliefs regarding group behavior therapy programs.

### Data analysis

The data were analyzed using SPSS 24.0 Version Statistical Software [21]. Percentages and frequencies were used to describe sociodemographic factors and knowledge. For attitude and beliefs, a standardized score was generated in SPSS with 1 point given for positive attitude and 0 points for negative attitude. If the total score was more than five, then, the participant had a positive attitude and positive beliefs, and if it was less than five, the participant had a negative attitude and beliefs. These scores were reported as the mean and standard deviation. One-way ANOVA was used to estimate the level of significance for the differences between the continuous variables and the chi-square test for categorical variables.

## Results

### Participants’ characteristics

The total number of completed questionnaires that were distributed in pre-defined smoking cessation in Riyadh was 229. Most of the participants aged between 21 and 40 years were well educated (Table 1).

**Table 1:** Sociodemographic characteristics for study participants

Characteristics	***N*** = 229 n (%)
**Age (Years)**	
≥ 18 - < 20	15 (6.6)
≥ 20 - < 30	81 (35.4)
≥ 30 - < 40	74 (32.3)
≥ 40 - < 50	31 (13.5)
≥ 50	28 (12.2)
**Educational level**	
Unable to read and write	6 (2.6)
High school or less	70 (30.6)
Bachelor	98 (42.8)
Diploma	37 (16.2)
Higher educational Degree	18 (7.9)
**Income (SR**^**a**^**)**	
≤ 5000	68 (29.7)
> 5000 - ≤ 10,000	69 (30.1)
> 10,000 - ≤ 15,000	63 (27.5)
> 15,000	29 (12.7)
**Job**	
Having job	184 (80.3)
Don’t have job	19 (8.3)
Student	26 (11.4)
**Residency**	
Riyadh	213 (93)
Others	16 (7)

^a^*SR* Saudi Riyals

Around 79% of participants had previously attempted to quit smoking, and most of them had more than two attempts (38.4%). About half of the participants smoked 10–20 cigarettes per day, and 30.1% of them had been smokers for 11 to 20 years (Table 2).

**Table 2:** Tobacco use behavior for study participants

Characteristics	***N*** = 229 n (%)
**Previous attempts to quit**	
Yes	181 (79)
No	48 (21)
**Number of attempts**	
Once	35 (15.3)
Twice	62 (27.1)
More than two	88 (38.4)
Never	44 (19.2)
**Type of tobacco**	
Cigarettes	177 (77.3)
Electronic tobacco	36 (15.7)
Chewing	1 (0.4)
Cigarettes and Electronic	12 (5.2)
Cigarettes and chewing	1 (0.4)
Chewing, Electronic and other	2 (0.8)
**Cigarettes per day**	
< 10 cigarettes	71 (31)
10–20 cigarettes	111 (48.5)
> 20 cigarettes	47 (20.5)
**Years of smoking**	
≤ 1	20 (8.70)
> 1 - ≤ 5	42 (18.3)
> 5 - ≤ 10	58 (25.3)
> 10 - ≤ 20	69 (30.1)
> 20	40 (17.5)

### Knowledge about group behavioral therapy for smoking cessation and its associated factors

Results from the current study showed a deficit in the knowledge about group behavior therapy (Table 3). Moreover, about half of them agreed to a certain degree that this type of treatment was appropriate to quit smoking. Having a high level of education was related to knowledge of group behavior therapy. The group in which 61.1% of people had higher education degrees knew the meaning of group behavior therapy. Therefore, the level of education was significantly associated with knowledge about group behavioral therapy (*p* = 0.04) (Table 4). There were no significant associations between age, income, previous attempts to quit smoking, number of cigarettes used per day, duration of years in tobacco use, and knowledge of group behavioral therapy.

**Table 3:** Knowledge about group behavior therapy programs for smoking cessation

Knowledge variable		***N*** = 229 n (%)
**1- Do you know what is group behavior therapy?**	Yes	81 (35.4)
	No	148 (64.6)
**2- Have you ever heard about these programs?**	Yes	89 (38.9)
	No	140 (61.1)
**3- Do you think this type of treatment is appropriate to quit smoking?**	Yes	66 (28.8)
	Yes, to certain degree	118 (51.5)
	No	17 (7.4)
	I don’t know	28 (12.2)

**Table 4:** Knowledge toward group behavioral therapy for smoking cessation according to different sociodemographic characteristics and tobacco use behavior

Variables	Do you know what is group behavior therapy? (yes) n (%)	***p*** values*	Have you ever heard about these programs? (yes) n (%)	***p*** values*
**Age (years)**				
≥ 18 - < 20	4 (26.7)	0.50	5 (33.3)	0.20
≥ 20 - < 30	29 (35.8)		33 (40.7)	
≥ 30 - < 40	31 (41.9)		35 (47.3)	
≥ 40 - < 50	10 (32.3)		8 (25.8)	
≥ 50	7 (25.0)		8 (28.6)	
**Education**				
Unable to read and write	2 (33.3)	0.04	2 (33.3)	0.08
High school or less	17 (24.3)		20 (28.6)	
Bachelor	39 (39.8)		43 (43.9)	
Diploma	12 (32.4)		13 (35.1)	
Higher educational Degree	11 (61.1)		11 (61.1)	
**Income (SR**^**a**^**)**				
≤ 5000	19 (27.9)	0.46	24 (35.3)	0.79
> 5000 - ≤ 10,000	27 (39.1)		29 (42.0)	
> 10,000 - ≤ 15,000	23 (36.5)		26 (41.3)	
> 15,000	12 (41.4)		10 (34.5)	
**Previous attempts to quit**				
Yes	64 (35.4)	0.10	68 (37.6)	0.44
No	17 (35.4)		21 (43.8)	
**Cigarettes per day**				
< 10 cigarettes	29 (40.8)	0.23	29 (40.8)	0.74
10–20 cigarettes	40 (36.0)		44 (39.6)	
> 20 cigarettes	12 (25.5)		16 (34.0)	
**Years of smoking**				
≤ 1	7 (35.0)	0.97	7 (35.0)	0.97
> 1 - ≤ 5	15 (35.7)		18 (42.9)	
> 5 - ≤ 10	20 (34.5)		22 (37.9)	
> 10 - ≤ 20	23 (33.3)		26 (37.7)	
> 20	16 (40.0)		16 (40.0)	

* *p* values from Chi-squared test, ^a^*SR* Saudi Riyals

### Attitude and beliefs toward group behavioral therapy for smoking cessation

**Fig. 1:**
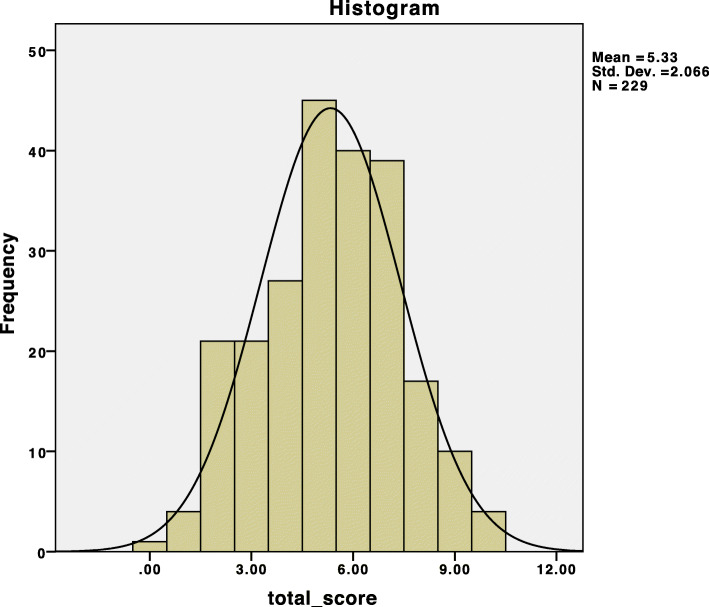
The distribution of the score for attitude and beliefs toward group behavioral therapy

The mean score for attitude and beliefs toward group behavioral therapy was 5.3 ± 2.06 (Fig. 1). Participants who had previous attempts to quit smoking showed a positive attitude and beliefs toward behavioral therapy (*p* = 0.05). In addition, smokers who consumed less than 10 cigarettes per day showed a positive attitude and beliefs toward behavioral therapy. This result implies that the number of cigarettes used per day influences the attitude and beliefs of the participants toward the group behavioral therapy (*p* = 0.03) (Table 5). Nevertheless, the association of sociodemographic factors and smoking behavior with attitudes and beliefs toward group behavioral therapy showed no significant association between age, level of education, income, years of smoking, and attitudes and beliefs.

**Table 5:** Association of sociodemographic characteristics with attitude and beliefs toward group behavioral therapy

Sociodemographic characteristics	Mean (SD)	F	*p*-value*
**Age (years)**			
≥ 18 - < 20	4.73 (2.31)	1.79	0.13
≥ 20 - < 30	5.35 (1.97)		
≥ 30 - < 40	5.14 (2.14)		
≥ 40 - < 50	5.26 (2.02)		
≥ 50	6.21 (1.93)		
**Education**			
Unable to read and write	3.67 (2.88)	1.10	0.10
High school or less	5.50 (1.88)		
Bachelor	5.12 (1.98)		
Diploma	5.86 (2.32)		
Higher educational Degree	5.28 (2.19)		
**Income (SR**^**a**^**)**			
≤ 5000	5.18 (2.05)	0.65	0.59
> 5000 - ≤ 10,000	5.35 (2.09)		
> 10,000 - ≤ 15,000	5.60 (2.16)		
> 15,000	5.07 (1.87)		
**Previous attempts to quit**			
Yes	5.52 (2.00)	7.76	< 0.05
No	4.60 (2.14)		
**Cigarettes per day**			
< 10 cigarettes	5.80 (2.12)	3.28	0.03
10–20 cigarettes	5.01 (2.11)		
> 20 cigarettes	5.38 (1.76)		
**Years of smoking**			
≤ 1	5.50 (2.40)	0.19	0.94
> 1 - ≤ 5	5.48 (2.16)		
> 5 - ≤ 10	5.31 (1.95)		
> 10 - ≤ 20	5.17 (2.03)		
> 20	5.40 (2.11)		

*p-value from one-way ANOVA test, ^a^*SR* Saudi Riyals

## Discussion

The findings confirm a deficit in knowledge about group behavior therapy. This might be due to the unavailability of this therapy approach in Saudi Arabia and the social barrier of smoking stigma in our community. Regarding the factors associated with knowledge deficit, it is clear that the level of education was associated with their knowledge. In addition, previous attempts to quit smoking and the number of cigarettes used per day, influenced the participants’ attitude and beliefs toward group behavioral therapy. Otherwise, there was no significant association between sociodemographic factors and smoking behavior with attitudes and beliefs toward group behavioral therapy.

However, a study found that smoking cessation success differed from country to country due to socioeconomic, personal, and political reasons. In addition, they found that age and income both have an association with success in smoking cessation. According to their study, as income increases the most positive effect on participants to quit smoking and success in cessation. In addition, as age increases, success in cessation increases, especially in the lower-education group [22].

It has been found that most of the respondents were young males; 79% of them had had attempted to quit smoking multiple times. In line with a previous study conducted in Makkah, 70.2% had tried at least once to quit smoking [23]. Another study that studied public knowledge and attitudes regarding smoking and smoking cessation treatments in New Zealand showed that 86% of the participants tried to quit smoking at least once [20]. Consequently, 78.3% of those who previously attempted to quit smoking started from one attempt to six attempts [18]. From these results, it is clear that the failure in smoking cessation programs could be a result of dependence on the pharmacological approach without any supportive intervention.

The current study revealed that the number of cigarettes used per day significantly influenced participants’ attitude and beliefs toward group behavioral therapy. Results showed that smokers consumed 10–20 cigarettes per day. The last survey conducted by the Saudi Health Interview Survey in 2020 showed the daily use of cigarettes with an average of 15 cigarettes per day [19]. This indicates a decrease in tobacco consumption compared to previous years and reflects the efforts of smoking cessation clinics. It is necessary to highlight the conclusion of prior research that smokers who consumed more than 20 cigarettes per day dropped out more frequently in the initial group meetings (*p* = 0.031) [24]. From this standpoint, the consumption of cigarettes might be a vital factor that can determine relapses during therapy.

Smoking habits tend to be acquired at an early age and are significantly associated with a high prevalence of smoking [3]. Similarly, as shown in our results, the average onset of smoking is between 11 and 20 years. In the current study, it has been found that the mean age of smokers was 35. Additionally, if we assume the onset of smoking 20 years ago, the smoking age would be almost 15 years, while the Saudi Health survey showed that the prevalence of smoking at the age of 15 years was 29 and 60.9% and that it started before 18 years [19, 25]. Therefore, this emphasizes the implantation of awareness programs at a young age, as suggested in a previous study in Saudi Arabia, that the preventive programs should started in primary schools before the age of 13 [26]. This suggestion is consistent with what has been found in a previous study that concludes the significance of prevention programs to reinforce non-smoking perceptions and behaviors after using a random clinical trial to assess the effects on smoking initiation and changes in beliefs [27]. It is critical to note that another study that showed that adult smokers older than 35 years were more likely to adhere to treatment (*p* = 0.017) than younger smokers [24]. This implies that most of the smokers who seek help are adults.

Smokers who want to quit had less knowledge than what we assumed about group behavior therapy programs. The reason behind this can be related to the lack of application of group behavior therapy programs since the programs are mainly dependent on the pharmacological approach with limited psychosocial interventions. Nevertheless, results showed that the participants’ level of education was significantly associated with their knowledge of this therapy approach. Although some participants were well educated, it did not affect their behavior. This finding was similar to that of a previous study [28].

In contrast, a study in Turkey found an association between level of education and success in smoking cessation. As the level of education had a greater positive effect on participants to quit smoking and progress in cessation. In addition, the higher the education level and age, the greater the success in cessation [22]. Hence, we suggest targeting the educated population while initiating these programs to obtain the ultimate results.

It is worth discussing these impressive results that previous attempts to quit smoking significantly influence the attitude and beliefs toward group behavioral therapy, which indicates that smokers encounter a serious problem with relapses. Additionally, the factors associated with success or failure to attempt smoking cessation showed that motivation is an essential factor in quitting smoking and maintaining cessation in the future [29, 30]. By reviewing the literature, many studies concur with the combination of pharmacological approaches with supportive intervention [7]. One study found an increase in chance from 70 to 100% to quit smoking [8]. In another review, a meta-analysis of 40 trials showed a significant benefit of the combination of pharmacological and behavioral therapy [31]. In addition, a study conducted on female prisoners showed that the combination of behavior therapy and pharmacological therapy was efficient compared to control groups [32].

Other studies have shown that smokers failed due to social reasons or friends’ influence (44%) [33]. This factor leads us to a critical question associated with the failure of quitting smoking. Does the smoker need to establish group behavior therapy programs to overcome the social impact?

### Strength and limitation

The strength of the current study is that it provides baseline information regarding knowledge, attitude, and beliefs toward group behavior therapy in our region. In addition, the study population was heterogeneous in terms of a previous attempt to quit smoking, which enriched the study findings. Furthermore, this study revealed the factors associated with the endorsement of a group behavior therapy program. On the other hand, the study has some limitations. The participants may not represent the Saudi population as the data were collected from five centers in Riyadh only. However, Riyadh is the capital city of the kingdom with a heterogeneous population from different socioeconomic classes. Moreover, the knowledge, attitude and beliefs toward group behavior therapy for smoking cessation were assessed only among male smokers. Lacking the relevant data on female might influence the findings. However, the prevalence of Saudi female smokers is low and they are less likely to attend the smoking cessation clinics compared to male. Another limitation is the scarcity of literature that assessed this topic internationally and the lack of previous studies in Saudi Arabia.

## Conclusion

This study demonstrated a deficit in the knowledge of people attending smoking cessation clinics regarding the group behavior therapy. Moreover, it showed that multiple factors play a role in smokers’ knowledge and attitudes, such as previous attempts to quit smoking, the number of cigarettes used per day, and the level of education. Although, The Ministry of Health in Saudi Arabia provided smoking cessation clinics in several regions around the country, these clinics mainly provide a pharmacological treatment. Group behavior therapy is not routinely provided as a modality of tobacco cessation assistance in these clinics. Despite the effectiveness of this method in smoking cessation, increase population awareness about the role of group behavior therapy in smoking cessation is required before this method is implemented in the routine practice.

## Supplementary Information

**Additional file 1.** The Questionnaire.

## Data Availability

The datasets used and analyzed during the current study are available from the corresponding author on reasonable request.
