# Supplementary material for: Knowledge, attitude, and beliefs toward group behavior therapy programs among male adults attending smoking cessation clinics, cross-sectional analysis
Source: BMC Public Health. 2021 May 5;21:868. doi: 10.1186/s12889-021-10924-4 (PMC8101190; doi:10.1186/s12889-021-10924-4)
Supplement: Supplementary file 1 — Additional file 1. The Questionnaire. [file 12889_2021_10924_MOESM1_ESM.docx]

**Supplementary file**

**Title: Knowledge, Attitude, and Beliefs Toward Group Behavior Therapy Programs Among Male Adults Attending Smoking Cessation Clinics, Cross-Sectional Analysis**

**Authors:**

Shatha A. Alduraywish ^1,2^, Meaad F. Alnofaie^3^, Balqes F. Alrajhi^3^, Fatima A. Balsharaf^3^, Sarah S. Alblaihed^3^, Alaa A. Alsowigh^3^, Wafa S. Alotaibi^3^, Fahad M. Aldakheel ^2,4^

**Affiliation:**

^1^ Department of Family and Community Medicine, College of Medicine - King Saud University, Riyadh - Saudi Arabia

^2^ Prince Sattam bin Abdulaziz Research Chair for Epidemiology and Public Health -, College of Medicine - King Saud University, Riyadh - Saudi Arabia

^3^ College of Medicine - King Saud University, Riyadh - Saudi Arabia

^4^ Department of Clinical Laboratory Sciences, College of Applied Medical Sciences, King Saud University, Riyadh, Saudi Arabia

**The Questionnaire**

| **Consent for participant in a study entitled**  “Knowledge, attitude and beliefs toward group behavioral therapy for smokers who want to quit smoking” |
| --- |
| **Purpose of study:**  To assess the Knowledge, attitude and beliefs toward “**group behavioral therapy**” for smokers who underwent or plan to quit smoking.  **Benefits of the study:**  Understanding the knowledge, attitude and beliefs of smokers who underwent or planned to quit smoking would benefit your treating specialist to incorporate this type of treatment in smoking cessation management and hence improve the care provided to smokers to support them quitting smoking.  **Possible Side effect:**  There will be no side effects associated with this study.  **Refuse to participate:**  If you refuse to participate, there will be no punishment.  **Confidentiality:**  This information will be confidential; data will be collected without showing your identity. This questionnaire requires around 10 minutes to complete.  **Primary investigator:** Dr.Shatha Alduraywish, Assistant professor, department of Family and Community Medicine, King Saud University  **Co-investigators:** Meaad alnofaie, Balqees Alrajhi, Fatima Balsharaf, Sarah Alblaihed, Alaa Alsowigh, Wafa Alotaibi  I agree to participate in this study |

| **Socio demographic** | **Response** |
| --- | --- |
| 1- How old are you? |  ≥ 18 - < 20   ≥ 20 - < 30   ≥ 30 - < 40   ≥ 40 - < 50   ≥ 50 |
| 2- What is your level of education? |  Unable to read and write   High school or less   Bachelor   Diploma   Higher educational Degree |
| 3- How much is your income? |  ≤ 5000   > 5000 - ≤ 10000   > 10000 - **≤** 15000   > 15000 |
| 4- What is your job? |  student   doctor   teacher   banker   shop seller   others   I don't work |
| 5- Where is your residency? |  Riyadh   Jeddah   Damam   Others. Where? …………….. |
| 6- Have you ever tried to quit smoking? |  Yes   No |
| 7- If Yes, How many attempts? |  Once   Twice   More |
| 8- the type of tobacco used |  Cigarettes   Electronic tobacco   Smokeless tobacco   Others |
| 9- How many cigarettes do you smoke a day? |  Less than 10 cigarettes   10 – 20 cigarettes   more than 20 cigarettes |
| 10- For how many years you smoke? |  ≤ 1 year   > 1 - ≤ 5 years   > 5 - ≤ 10 years   > 10 - ≤ 20 years   > 20 years |

| Questions that are related to your knowledge about group behavior therapy | Response |
| --- | --- |
| 11- Do you know what is group behavior therapy? |  Yes   No |
| 12- Have you ever heard about these programs? |  Yes   No |
| 13- Do you think this type of treatment is appropriate to quit smoking? |  Sufficient   Sufficient to certain degree   Not sufficient |

Group behavioral therapy

- It is including one specialist or more to lead the session with one group that have (5 -15) members who are suffering from the same issue.
- The aim of these session is to support participants to share their ideas and emotions toward their experience.
- The duration is one to two hours a week.
- The number of the session are 10-20 sessions and it might need more if needed.

| Questions about your attitude and believes toward behavioral group therapy? | Response |
| --- | --- |
| 14- How much do you like this type of therapy? |  I like it   I like it to some extent   I do not like it |
| 15- How much do you think there will be risks in this type of therapy? |  There is no risk   There will be little risk   There are a lot of risks |
| 16- Do think this type of therapy is effective? |  Effective   Effective to some extent   Not effective |
| 17- What is the probability of this type of therapy to make you quit smoking permanently? |  Probable   There is a probability to some extent  Improbable |
| 18- How much do you think about occurrence of side effects when you use this type of therapy? |  There is no side affect   There will be a little side affect   There are a lot of side affects |
| 19 - How much you will be discomfort by using this type of therapy? |  There is no discomfort   There will be a little discomfort   There is much discomfort |
| 20-This therapy cause social stigma? |  Agree   Neutral   Disagree |
| 21- The therapy will be costly? |  Agree   Neutral   Disagree |
| 22- The therapy will require me a huge effort? |  Agree   Neutral   Disagree |
| 23- The therapy will require me to be committed? |  Agree   Neutral   Disagree |
| 24- Generally, what is your opinion toward this type of therapy? |  Positive   Neutral   Negative |

| Research about Knowledge, attitude and beliefs toward group behavioral therapy for smokers who want to quit smoking. |
| --- |
| Thank you. |
| Thank you for your participating in the study. The aim of the study is to measure your Knowledge, attitude and beliefs toward group behavioral therapy for smokers who want to quit smoking. |
